# Accentuated osseointegration in osteogenic nanofibrous coated titanium implants

**DOI:** 10.1038/s41598-019-53884-x

**Published:** 2019-12-09

**Authors:** Siddhartha Das, Kanchan Dholam, Sandeep Gurav, Kiran Bendale, Arvind Ingle, Bhabani Mohanty, Pradip Chaudhari, Jayesh R. Bellare

**Affiliations:** 1 Department of Biosciences and Bioengineering, Indian Institute of Technology Bombay, Mumbai, 400076 Maharashtra India; 2 Department of Chemical Engineering, Indian Institute of Technology Bombay, Mumbai, 400076 Maharashtra India; 3 Department of Dental and Prosthetic Surgery, Tata Memorial Centre, HBNI, Mumbai, 400 012 Maharashtra India; 4 Advanced Centre for Treatment, Research and Education in Cancer, Navi Mumbai, 410 210 Maharashtra India; 5 Wadhwani Research Centre for Bioengineering, Indian Institute of Technology Bombay, Mumbai, 400076 Maharashtra India

**Keywords:** Mesenchymal stem cells, Fixed prosthodontics

## Abstract

Anchoring of endosseous implant through osseointegration continues to be an important clinical need. Here, we describe the development of superior endosseous implant demonstrating enhance osseointegration, achieved through surface modification via coating of osteogenic nanofibres. The randomized bio-composite osteogenic nanofibres incorporating polycaprolactone, gelatin, hydroxyapatite, dexamethasone, beta-glycerophosphate and ascorbic acid were electrospun on titanium implants mimicking bone extracellular matrix and subsequently induced osteogenesis by targeting undifferentiated mesenchymal stem cells present in the peri-implant niche to regenerate osseous tissue. In proof-of-concept experiment on rabbit study models (n = 6), micro-computed tomography (Micro-CT), histomorphometric analysis and biomechanical testing in relation to our novel osteogenic nanofibrous coated implants showed improved results when compared to uncoated controls. Further, no pathological changes were detected during gross examination and necropsy on peri-implant osseous tissues regenerated in response to such coated implants. The findings of the present study confirm that osteogenic nanofibrous coating significantly increases the magnitude of osteogenesis in the peri-implant zone and favours the dynamics of osseointegration.

## Introduction

Implants made of commercially pure titanium and titanium alloys are most widely accepted and successfully used due to their favourable combination of biocompatible features^1^, corrosion resistance^2^ and an unique intrinsic propensity to osseointegrate^3^. Osseointegration was originally defined by Brånemark, as a “direct structural and functional connection between ordered living bone and the surface of a load-carrying implant”^4,5^. In other words, osseointegration involves the incorporation of nonvital component in the living bone leading to an efficient, reliable and predictable anchorage mechanism^6^. In order to successfully osseointegrate, an implant should have a firm and immovable connection (without micro-motion in the osseous site) between the implant biomaterial surface and the surrounding osseous tissue known otherwise as primary stability^7^. The favourable treatment outcome of titanium implants, *in vivo*, is also based on bio-response of these implants to osteogenic cells i.e. osteoblasts during the healing period^8^. However, in spite of all these advantages, titanium implants has its own sets of limitation and is described elsewhere^9,10^. Hence, in order to promote bioactivity and enhance bone integration, numerous methods related to surface modifications or coatings of titanium implants are being studied extensively. Surface modifications such as titanium plasma – spraying^11^, grit – blasting^12^, anodization^13^, acid-etching^14^ or calcium phosphate coatings^15^ are widely investigated upon. Yet, further research on peri-implant tissue response to novel coatings or surface modification of implants is warranted as the optimal dynamics of implant osseointegration still remains elusive.

Furthermore, the healthy bone, a type of heterogeneous tissue, that incorporates the titanium implant comprises of bone extracellular matrix (ECM), secreted mostly by osteoblasts^16^. The bone ECM are made of numerous ECM proteins like proteoglycans, glycosaminoglycans, glycoproteins, collagen type I, non-collagenous proteins, fibronectin etc.^17^ and regulates cell - matrix interactions including cross-talk between osteoblasts and osteoclasts^16^, influences the differentiation of mesenchymal stem cells^18,19^ and serves as a critical platform upon which mineralization (in bone) takes place^20^.

Currently, various tissue-engineering approaches with bioactive scaffold are being explored to regenerate osseous tissue by directing mesenchymal stem cell differentiation to osteogenic lineages. Interestingly, artificially fabricated electrospun nanofibrous scaffold mimics the form and function of native ECM^21,22^. The 3D porous electrospun nanofibrous scaffold exhibits high surface-to-volume ratio and facilitates in nutrient transport, cell binding, adhesion, migration, proliferation and differentiated function^23^.

**Figure 1:**
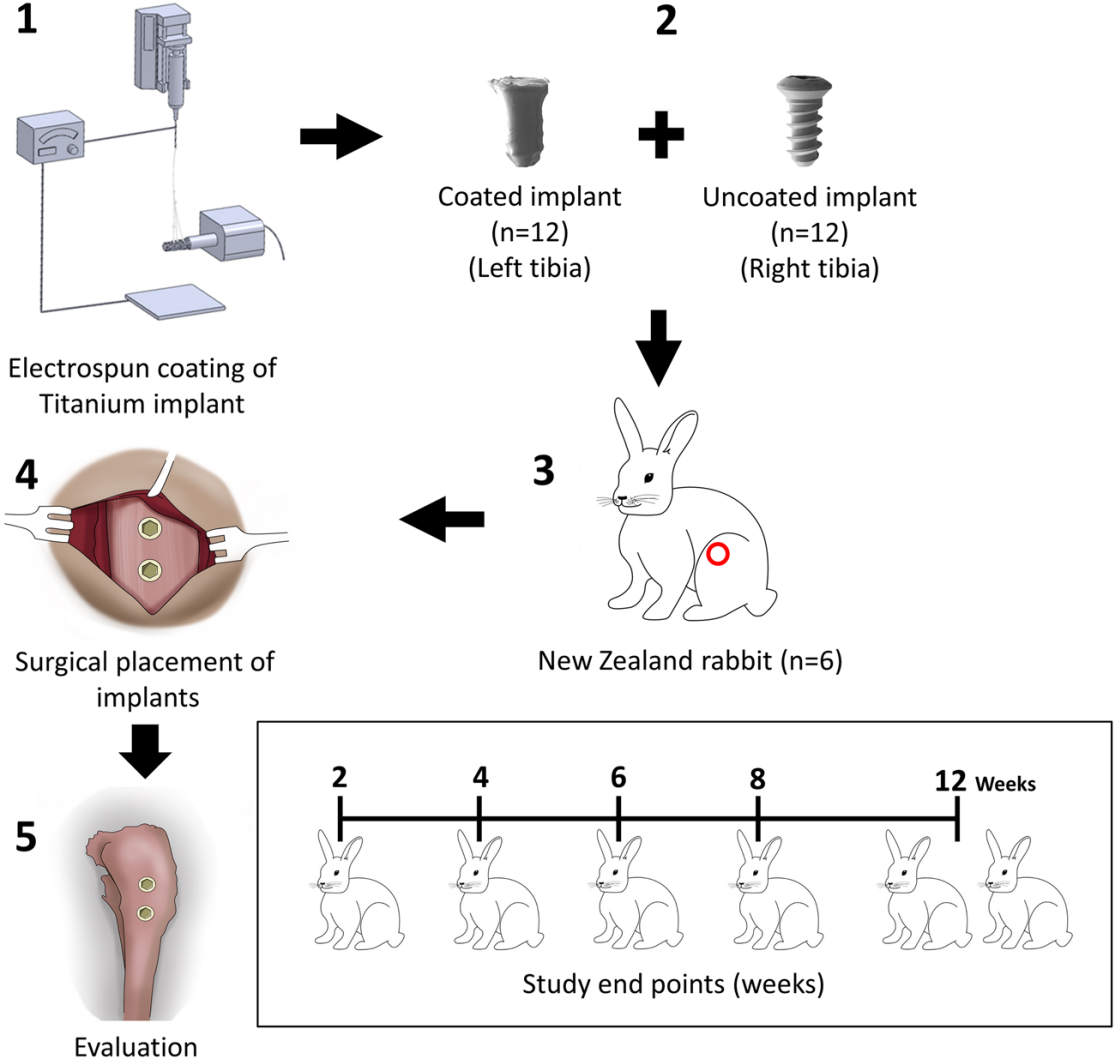
Experimental design *in vivo*. The osteogenic nanofibres were fabricated on the surface of screw-type titanium implants by modification of electrospinning apparatus. All rabbits (n = 6) received 4 implants – 2 coated and 2 uncoated implants in left and right tibia respectively. The temporal assessment of regenerated osseous tissue in response to respective implants was performed at the end of 2^nd^, 4^th^, 6^th^, 8^th^ and 12^th^ week.

**Figure 2:**
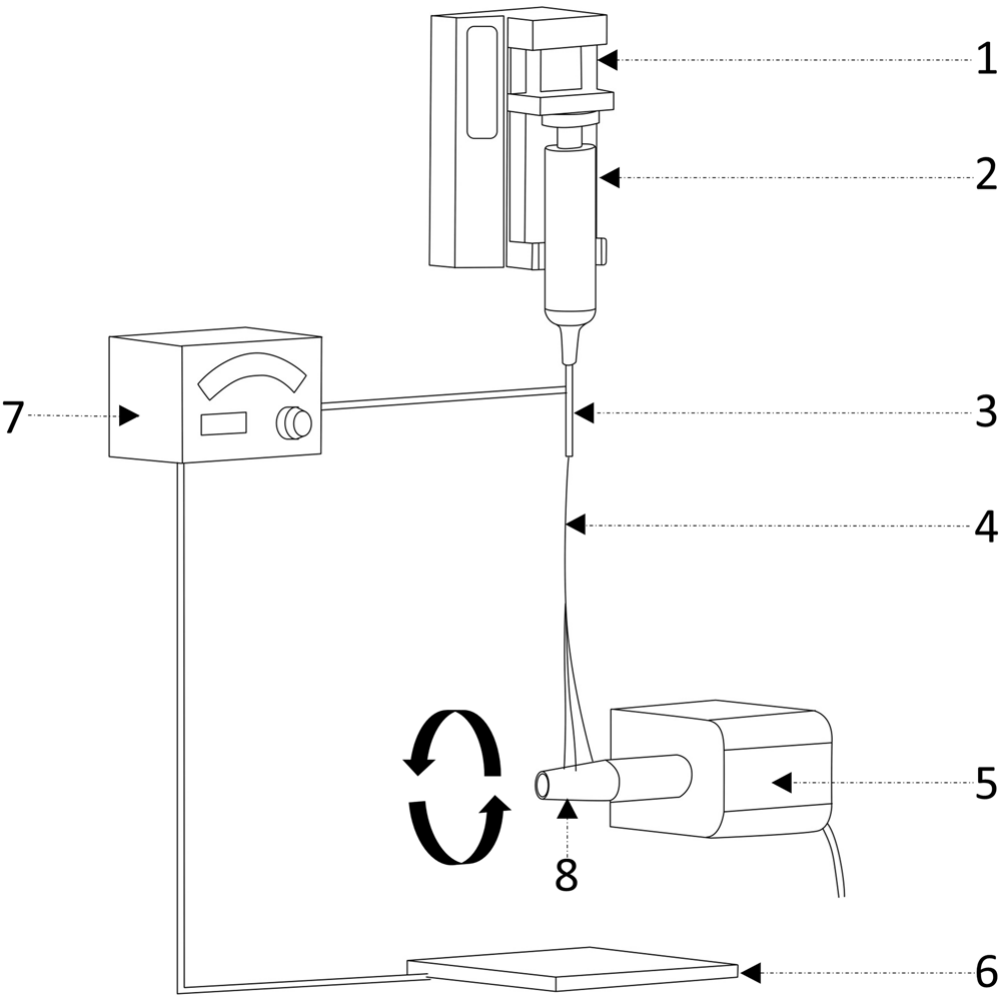
Schematic diagram for fabrication of osteogenic nanofibres on the surface of titanium implant. 1—syringe pump, 2—syringe loaded with polymer solution, 3—needle, 4—nanofibres, 5—DC motor, 6— collector plate, 7—voltage generator, 8— titanium screw attached to the shaft of the motor.

Our preliminary *in vivo* study has confirmed the bioactivity of our novel osteogenic nanofibrous coatings on titanium implants^24^ further demonstrating our attempt to fabricate an ideal implant surface^8,24–26^. In the present investigation, we have performed an elaborate and detail evaluation of osteogenic nanofibrous coated titanium implants on rabbit study models (Fig. 1). The nanofibrous coating around the titanium implant (Fig. 2) mimics ECM and possesses osteoinductive and osteoconductive features^24^. We hypothesize that the osteogenic nanofibrous coating on titanium implant (Fig. 2) will act as an artificial ECM in the peri-implant zone and will provide a 3D *in vivo* microenvironment for mesenchymal stem cells in the peri-implant region to grow, proliferate and differentiate to osteogenic lineage resulting in enhanced, improved and rapid osseointegration.

## Results

### ESEM (Environmental scanning electron microscopy) and FIB-SEM (Focused ion beam scanning electron microscopy) analysis of the coated and uncoated implants

**Figure 3:**
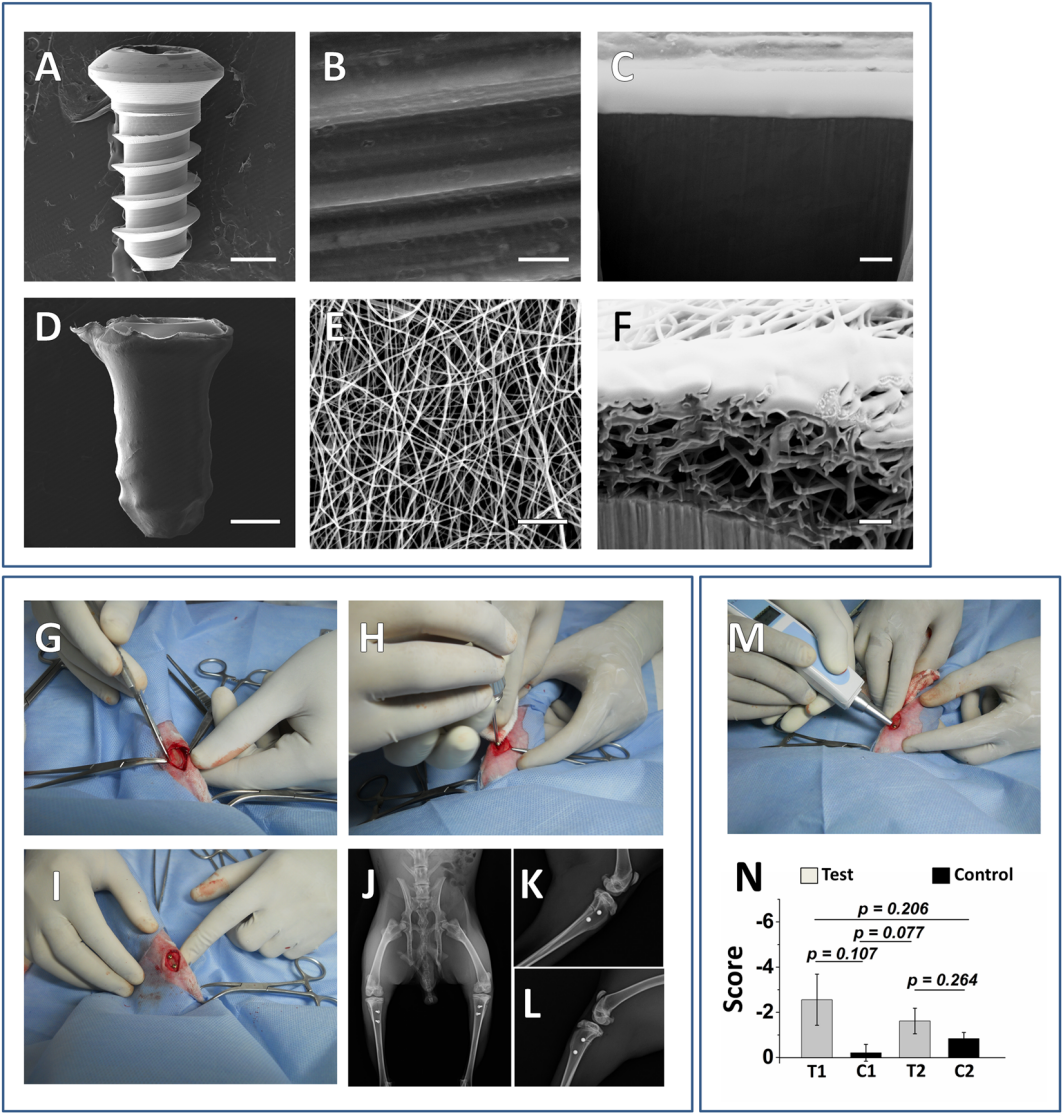
SEM, FIB-SEM images showing the topographical difference between uncoated and nanofibrous coated implants, demonstration of surgical procedure and measuring the quality of osseointegration in implants by Periotest device (**A**) low magnification (40X) of uncoated titanium implant, **(B)** high magnification (10,000X) of uncoated titanium implant, (**C**) FIB-SEM images of cross sectional view of uncoated titanium implant. Protective platinum layer is seen immediately above the TiO_2_ layer and bulk titanium substrate. (**D**) low magnification (40X) of nanofibrous coated titanium implant, (**E**) high magnification (10,000X) of nanofibrous coated titanium implant. The nanofibres, fabricated on the surface of the coated titanium implant completely obscure the microstructures of uncoated machined titanium implant. (**F**) FIB-SEM images of cross-section of titanium implant coated with osteogenic nanofibres. The stacks of nanofibres appeared to be adherent on the surface of titanium substrate. Image scale bar represents: A, D - 1 mm; B, E - 5 µm and C, F – 1 µm. Representative images of surgical procedure **(G–I)** with post-operative high resolution anterior-posterior **(J)** and medio-lateral radiographs **(K**,**L)** were obtain for each tibia for all rabbits, to confer the real position of implants, qualitatively assessing bone regeneration, implants orientation along with its alignment and to rule out complications associated with implant surgery and healing in the receptor animal models. Periotest device for measuring the quality of osseointegration in implants. **(M)** Each limb of rabbit was stabilized and the Periotest device was placed at an angle of ~45° for percussing the osseointegrated implant and record the Periotest values (PTVs). **(N)** Mean PTVs. Abbreviations: - T1 - Test implant no. 1, - T2 -Test implant no. 2, - C1 - Control implant no. 1, - C2 - Control implant no. 2. Data are expressed as mean ± standard error of mean (SEM). Student’s t-test was performed with data significance indicated with (*) for p < 0.05.

ESEM and FIB-SEM images reveals major differences in the surface topography between the uncoated and nanofibrous coated titanium implant (Fig. 3A–F). The coated implant could be easily distinguishable from the uncoated one at all magnifications due to significant changes in its surface topography by virtue of nanofibrous coating. In uncoated titanium implants, the machining process induced anisotropic features containing ridges and valleys in the form of unilateral striations appeared relatively smooth at higher magnifications (10,000X) (Fig. 3B). These anisotropic features were concealed by randomly oriented nanofibrous coating, and hence the patterns were no longer evident on coated screws. At 10,000 X magnification, the coated titanium implant (Fig. 3E) exhibited bead-free randomly aligned nanoscaled fibres with interconnected pores that formed under controlled parameters^24^ (applied solution voltage, feed rate and tip-to-collector distance were 15 kV, 0.34 ml h^−1^ and 13 cm respectively) with mean fibre diameters of 186.62 ± 22.60 nm. The FIB based cross-sectional photomicrograph of the titanium implant reveals the protective platinum layer followed by an intermediate layer of titanium oxide which covers the pure titanium implant. The findings correspond with the results of similar studies^27–29^. The cross-sectional morphology of the osteogenic nanofibrous coated titanium implant is shown in Fig. 3F. The layer of osteogenic nanofibrous mat covered by protective platinum layer gives the impression of close approximation of nanofibres with the surface of titanium implant via a layer of titanium oxide. The image further reveals that the diameter of individual nanofibres are in the range of 185.79 ± 17.32 nm and are in accordance with our ESEM findings. Additionally, the nanofibres mostly exhibited solid circular/partially collapsed ribbon like fibres and their arrangement are in the form of continuous stacks over the implant surface. Judging by the nature of deposition of fibres, it is obvious that the fibres starts coating from the surface of implant and proceed upwards and outwards. Hence, the crest of the titanium implant displayed thinner deposition of nanofibrous coating when compared to base of the thread and its respective flanks. Surgical steps (Fig. 3G–I) and immediate post-operative radiograph (Fig. 3J–L) were evaluated to determine the accuracy of implant placements.

### Periotest for assessment of the osseointegration

Out of 24 implants (12 tests and 12 controls), 19 implants were subjected to periotest evaluation (Fig. 3M). For negating bias, the periotest values (PTVs) obtained in early events of osseointegration i.e. the implants (n = 4) placed in rabbit no.1 (euthanized in 2^nd^ week) were not taken into consideration. Further, a control implant (implant no. 2) in rabbit, euthanized on 4^th^ week could not be percussed due to formation of proliferating fibrous mass surrounding the head of screw type implant. The mean PTVs were negative for 18 implants (includes both test and control) and positive for a single control implant placed in rabbit no. 2, euthanized on 4^th^ week of study period (Supplementary Fig. 1). The mean PTVs were significantly lower in test implants (−2.08 ± 0.47) compared to controls (−0.52 ± 0.31) (Fig. 3N). Further, it was noted that the values of PTVs in test implants decreased significantly with increase in duration of study end points (Supplementary Fig. 1).

### Macroscopic grading of implant site

**Figure 4:**
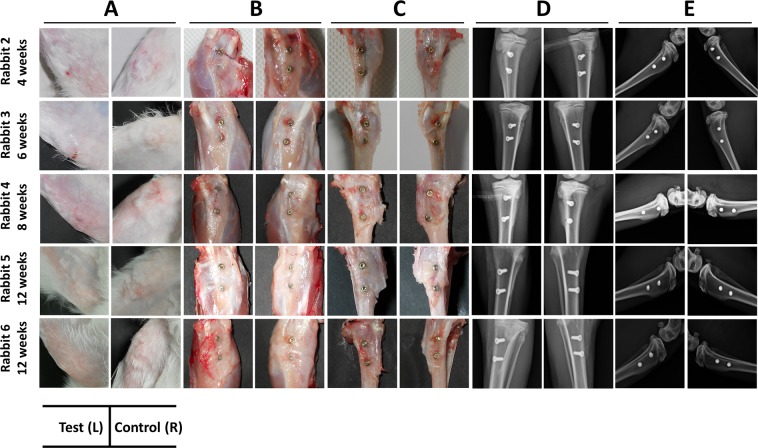
Macroscopic and radiographic images of healed tibia at the end of respective time points. (**A**) Gross/macroscopic images of skin around implant and peri-implant region, (**B**) subcutaneous region after excision of skin, (**C**) exposed implants after excision of muscles and soft tissues, (**D**) anterior-posterior radiograph of tibiae, (**E**) medio-lateral radiograph of tibiae. The test implants have not exerted any untoward influence on peri-implant area, as suggested by absence of discolouration, osteolysis and rejection reaction etc. Hence, no complications in healing or occult defects with respect to these implants were noted.

In the post-operative period, all rabbits bore the weight on operated limbs without any fixative/supportive devices. At necropsy, gross examination revealed normal postoperative healing for all 6 rabbits (Fig. 4A). Further, examination of subcutaneous tissue (Fig. 4B), after dissection of muscles and deep fascia (Fig. 4C) and post-operative radiographs (Fig. 4D,E) revealed no significant findings. No signs of implant loosing or signs of infections like pus/abscess formation or ulceration were noted. Palpation of popliteal lymph nodes revealed absence of localised lymphadenopathy. Macroscopically, the newly formed peri-implant bone appeared normal. The peri-implant bone of all test implants in animals appeared normal (macroscopic grade 0) except in the left tibia of rabbit no. 3 (macroscopic grade 1) which showed a region of ~0.5 mm hyperplastic fibrous mass emanating from the bone-implant interface (implant no 2). Additionally, rabbit no.1 completing 2 weeks of study period was excluded from the grading system as it elicited early healing events in the peri-implant zone and may influence adversely the grading scores. A summary of the macroscopic objective assessment of peri-implant bone for test and controls are presented in the Table 1 below.

**Table 1:** Summary of peri-implant tissue response with respective scores for each implant, osseointegrated in proof of concept trial in animal models.

Period of observation	Test Implants	Score	Control Implants	Score	Observation/Findings
2 weeks (Rabbit No. 1)	Implant no. 1	—	Implant no. 1	—	—
	Implant no. 2	—	Implant no. 2	—	—
4 weeks (Rabbit No. 2)	Implant no. 1	0	Implant no. 1	0	Unremarkable morphology
	Implant no. 2	0	Implant no. 2	2	Moderate hyperplastic mass on peri-implant area of control implant (no. 2)
6 weeks (Rabbit No. 3)	Implant no. 1	0	Implant no. 1	1	Slight hyperplastic mass on peri-implant area of control implant (no. 1)
	Implant no. 2	1	Implant no. 2	0	Slight hyperplastic mass on peri-implant area of test implant (no. 2)
8 weeks (Rabbit No. 4)	Implant no. 1	0	Implant no. 1	0	Unremarkable morphology
	Implant no. 2	0	Implant no. 2	2	Moderate hyperplastic mass on peri-implant area of control implant (no. 2)
12 weeks (Rabbit No. 5)	Implant no. 1	0	Implant no. 1	0	Unremarkable morphology
	Implant no. 2	0	Implant no. 2	1	Slight hyperplastic mass on peri-implant area of control implant (no. 2)
12 weeks (Rabbit No. 6)	Implant no. 1	0	Implant no. 1	1	Slight hyperplastic mass on peri-implant area of control implant (no. 1)
	Implant no. 2	0	Implant no. 2	1	Slight hyperplastic mass on peri-implant area of control implant (no. 2)

### Micro-CT evaluation

**Figure 5:**
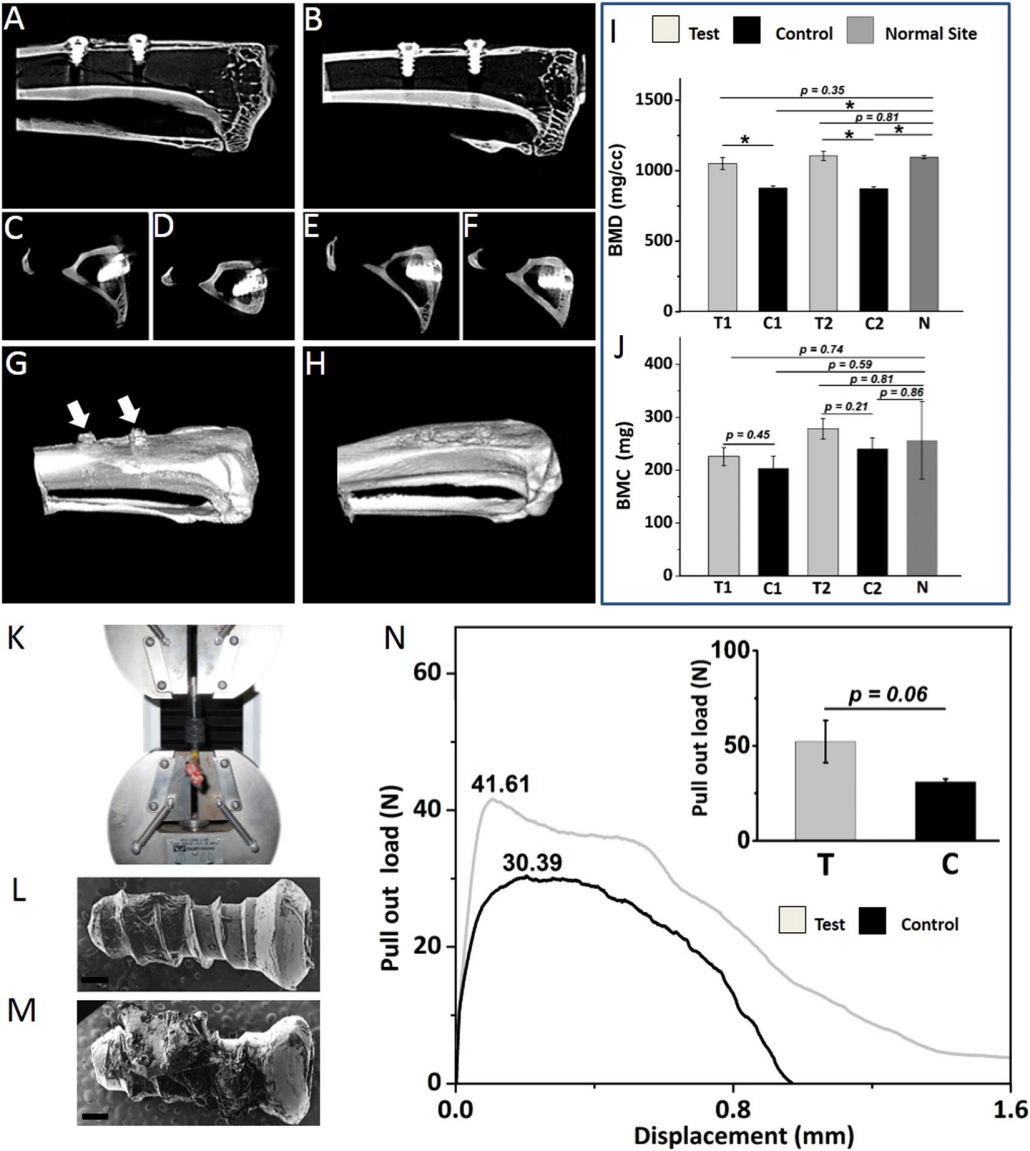
Analysis of trabecular bone determined by micro-CT and pullout evaluation procedure in test and control implants placed in rabbit’s tibiae at the end of 12^th^ week. (**A**) cross section of right tibia with control implants in sagittal plane (**B**) left tibia with test implants in sagittal plane (**C**) cross section of control implant no. 1 in axial plane (**D**) control implant no. 2 in axial plane (**E**) test implant no. 1 in axial plane (**F**) test implant no. 2 in axial plane. 3D images of (**G**) right and (**H**) left tibia of rabbit at the end of 12^th^ week post implantation. The titanium implant screws has been omitted from the 3D images. The presence of hyperplastic fibrous tissues emanating upwards and outwards from the bone-implant interface could be noted surrounding the control implants (arrows).The cortical bone surrounding the test implants demonstrates significantly greater values over time in BMD (**I**), BMC (**J**) relative to controls and are comparable to “normal” unoperated sites. (**K**) Procedure of mechanical pull-out tests of osseointegrated implants. Representative SEM image of the retrieved (**L**) control implant and (**M**) test implant. Scale bar represents 200 μm (**N**) Representative Force–displacement curves for the test and control implant after 12 weeks study period. The figure inset displays the mean result of ultimate tensile strength for test and control implants. The test implant exhibited increase residual calcified tissues on its surface and higher ultimate tensile strength compared to control. Abbreviations:- BMD - Bone mineral density, - BMC - Bone mineral content, -T- Test implants, -C- Control implants, - T1 - Test implant no. 1, - T2 -Test implant no. 2, - C1 - Control implant no. 1, - C2 - Control implant no. 2,- N - Normal (unoperated) site of tibia. Data are expressed as mean ± standard error of mean (SEM). Student’s t-test was performed with data significance indicated with (*) for p < 0.05.

Micro-CT 2D (in sagittal and axial planes) and 3D images (representative images of 12^th^ week rabbit’s tibia) demonstrates spontaneous regeneration of bone at the implant-bone interface for both type of implants (Fig. 5A–H). Upon closer inspection, the regenerated osseous tissue with immediate contact on the surface of test implants exhibited less radiolucent gaps and increased homogeneous radiopacity. Further, the regenerated osseous tissue in contact with test implants displayed higher bone mineral density (BMD) (1078.63 ± 26.92 for tests and 875.39 ± 2.23 for controls) and bone mineral content (BMC) (252.14 ± 26.20 for tests and 222.37 ± 18.64 for controls) which was 23.22% and 13.39% higher than control implants respectively (Fig. 5I,J). Radiographically, the proliferation of newly mineralized tissue appeared to be guided by the surface of the test implant resulting in a higher grade of bone integration. Further, improved values were noted for interfacial cortical plate thickness and Implant Bone Integrated Volume (IBIV) in both axial and sagittal plane for test implants when compared to controls (Supplementary Fig. 2).

### Biomechanical evaluation: Pull-out test

Pull-out tests were performed successfully on 6 specimens (Fig. 5K). Comparisons were made between the respective test and control implants (Fig. 5L–N) located in contralateral position in tibia to negate variation(s) in cortical bone thickness surrounding the implant in rabbits euthanized at the end of respective study period. A significantly greater pullout strength was noted for test-implants (n = 3) in comparison to controls (n = 3) (representative image, Fig. 5N). The mean result of ultimate tensile strength for test implants (52.15 N) was significantly higher when compared to controls (30.90 N) (inset, Fig. 5N). The coating on the test provided a 68.77% increase in pullout strength when compared to uncoated control. Further, the demonstration of extensive residual bone tissue noted on the surface of retrieved test implants (representative image, Fig. 5M) confirms that the interfacial strength of test implant was significantly higher than that of control.

### Histological and histomorphometric evaluation

**Figure 6:**
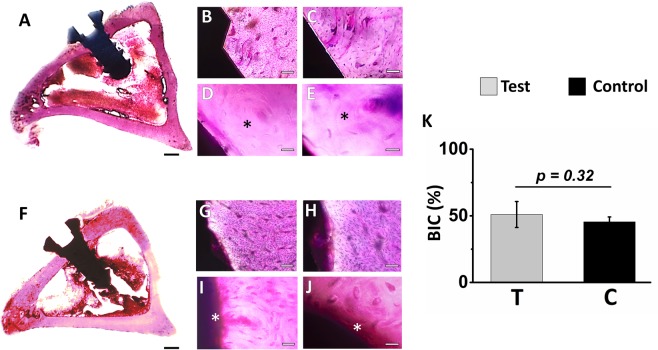
Histological photomicrograph of implant bone specimen stained with Hematoxylin & Eosin and bone-implant contact percentage of test and control implants after 12 weeks. Representative images of rabbit’s tibia in axial plane (**A**) test implant with magnified view (**B**–**E**) showing mature bone (*) in contact with implant surface, (**F**) control implant with magnified view (**G**–**J**) showing the presence of osteoid seam (*) on implant surface. Lamellar bone deposition on the surface of test implant is observed resulting in dense and intimate apposition of bone on the implant surface, (**K**) bone–implant contact percentage for test and control implants. Abbreviations: - T- Test implant, -C- Control implant. Scale bar for A and F represents 1 mm, scale bar for B and G represents 100 μm, scale bar for C and H represents 50 μm, scale bar for D and I represents 20 μm, scale bar for E and J represents 10 μm. Data are expressed as mean ± standard error of mean (SEM). Student’s t-test was performed with data significance indicated with (*) for p < 0.05.

On lower magnification, undecalcified ground thin histological sections (in axial plane), the endosseous part of titanium implants (test and control) are observed extruding the healed cortical plates of tibia, resulting in protrusion of these into the marrow cavity without any communication with the opposite cortices, thereby appeared to be precisely inserted into the cortical and spongy bone canal, further not affecting cells in the marrow cavity. Loose connective tissues with normal cellular architecture were observed surrounding the intraosseos part of implants. At higher magnification, the interface tissue of test implants by the end of 2^nd^ week exhibited increased osteoblastic activity and prominent Volkman’s canal suggesting increased vascularization in the peri-implant zone compared to controls. At the end of 4^th^, 6^th^ and 8^th^ week, histological analysis of regenerated osseous tissue of test and control implants reveals that osteoid seam was in the process of gradual maturation. At 12^th^ week, well organized and mature secondary osteon were noted on regenerated bone in close proximity to titanium implant surface for both test and controls (Fig. 6A–J). However, increased areas of less organized osseous tissues were noted in regenerated areas close to control implants (Fig. 6G–J). On the other hand, significant proliferation of osseous tissue was observed over the entire outline of test implants. Interestingly, the peri-implant zone of test implant revealed an assemblage of bony trabeculae with no or very few unmineralized region. In case of control implants, the bone matrix prominently demonstrates layers of osteoid secreted by functional osteoblasts suggesting an increasing production of woven bone which are in the process of being remodelled to mature lamellar bone (Fig. 6G–J). Notably, at the end of study period, the bone-implant-contact region, in test implants (50.90 ± 9.75%) demonstrates extensive secondary osteon with Volkman’s canal suggesting an early maturation and vascularization of osseous tissue induced by the osteogenic nanofibrous coating compared to controls (45.43 ± 3.67%) (Fig. 6K). Further, complete dissolution of bioactive coating was noted on the surfaces of test implants. Remnants of dead bone fragments, inflammatory response, rejection reaction, infections etc. at the interface of test implants were absent.

## Discussion

**Figure 7:**
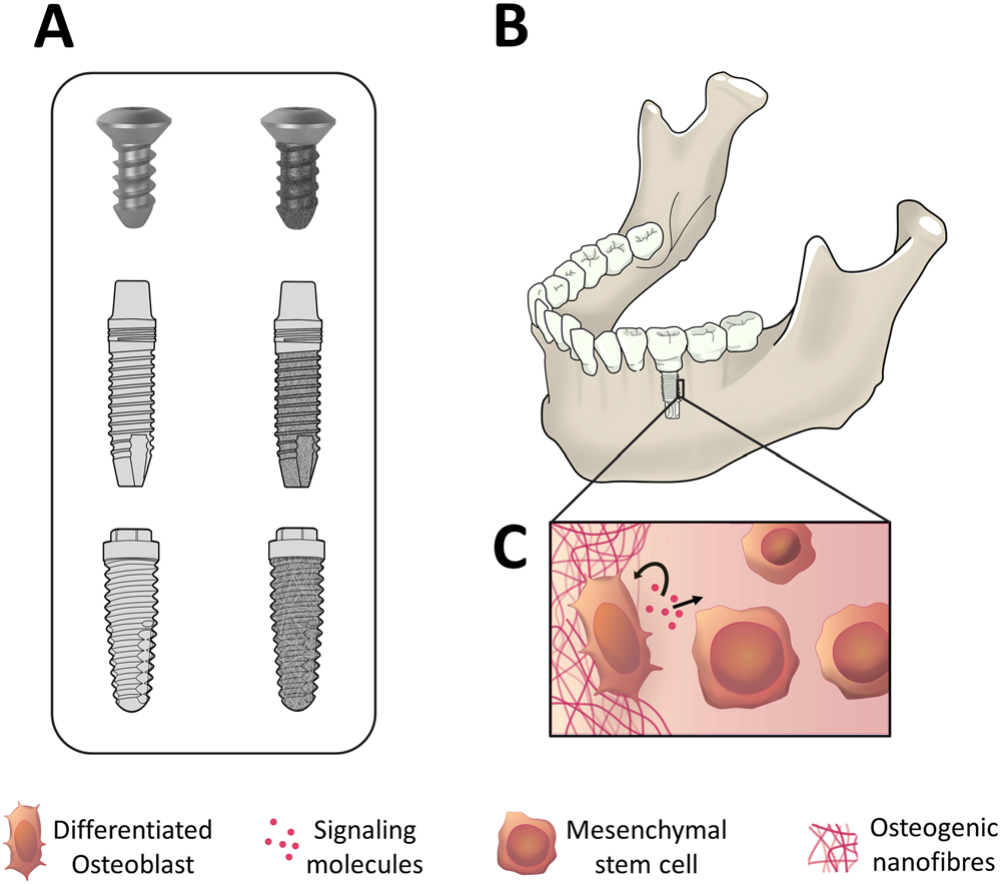
Clinical applications of osteogenic nanofibrous coating and its mechanism of action. (**A**) Our invention will be relevant for coating titanium implants of various geometries (e.g. bone screws, dental implants with cylindrical/conical/straight/hybrid design etc.) (**B**) Osteogenic nanofibrous coated titanium dental implant placed in the edentulous region of mandible (**C**) Autocrine and paracrine signaling of MSCs in the peri-implant zone by virtue of our nanofibrous coating will result in enhanced regeneration of bone and better osseointegration of implants.

In spite of recent advances, the ideal surface modification of titanium implant for optimal osseointegration is yet an unresolved issue^30,31^. In humans, a titanium implant generally involves a prolong period of 3–4 months to integrate with bone^32–34^. Moreover, it has been claimed that regeneration of osseous tissue in rabbit study models are rather swift and all events related to healing may span for a period of approximately six weeks^32,35^. Thus, in the present study, an efficient, economical and reproducible method of fabricating osteogenic nanofibrous coating on the surface of titanium implant (Fig. 7A) and its subsequent *in vivo* bio-response in rabbit models have been explored. To our knowledge, this is the first detail and elaborate *in vivo* study to establish the correlation between the coating of osteogenic nanofibres around the titanium implant surface and its subsequent effect in prompt *in vivo* osseointegration. The distinctive character of such coated implants is the successful fabrication of biodegradable ultrafine osteogenic nanofibres, mimicking extracellular matrix which provides stem cell niche like regions^36,37^. These regions are favourably utilized by mesenchymal stem cells for attachment, growth, proliferation and differentiation resulting in promotion of osseointegration. Such features, like highly porous and interconnected mesh with large surface-to-volume ratio coupled with systemic tackling of associated intrinsic limitations are otherwise difficult to achieve in conventional metallic implants^38,39^. The surface of the titanium implants were coated by modifying the electrospinning setup^24^. The osteogenic nanofibrous coated implants was suggested to promulgate more porous and interconnected surface layer in contrast to commercially used implants^24^. Here, we have attempted to provoke the MSCs in rabbits’ tibia analogous to rich source of MSCs residing in maxillofacial region (Fig. 7B) into osteoblastic lineages for regeneration of osseous tissue^40,41^.

Besides that, literature suggests interesting facts about the components included in the nanofibres used for coating titanium implants e.g. dexamethasone involves two types of mechanism for differentiating MSCs into osteoblastic lineages^24^. Recently, it was reported that under the influence of dexamethasone, MSCs differentiates into osteoblasts due to activation of WNT/β-catenin signaling pathway^42^. An alternate mechanism might be dexamethasone induced modulation of RUNX 2 phosphorylation^43^. While the production of Col 1 in ECM is favourably influenced by Ascorbic acid^44^. Additionally, β-Glycerophosphate, a source of phosphate for hydroxyapatite, aids in phosphorylation of ERK1/2 signaling pathway^45^. Furthermore, reports confirms the osteoconductive potentials of Hydroxyapatite^46,47^. Hence, a strategy that enables enhance osseointegration by virtue of promotion of neo-bone formation should not only promote osteoinduction but also osteoconduction is being formulated. Thus, we have incorporated both i.e. osteoinductive and osteoconductive chemicals to fabricate bead free, uniform, randomly aligned osteogenic nanofibres on the surface of titanium implants. The promotion in osseointegration of titanium implants is related to three principal factors: (1) The osteogenic nanofibrous coating mimics native environment of the stem cell niche in bone and provides favourable attachment sites for stem cells present in the peri-implant zone to grow, proliferate and differentiate (2) The osteoinductive chemicals present in the nanofibres induces osteogenic differentiation of multi-potent stem cells (3) Osteoconduction results in new bone formation and direct bone apposition on the implant surface. Additionally, the *in vivo* physiological environment might render osteogenic chemicals incorporated in the biodegradable nanofibres to be delivered locally in the peri-implant area thereby initiating osteogenesis and regeneration of osseous tissue.

As mentioned earlier, all implants (n = 24) achieved successful bone integration and demonstrated secondary stability at the end of their respective study points (i.e. 2,4,6,8 and 12 weeks) post implantation. The absence of peri-implant radiolucency further affirms our perspective. Macroscopically, uneventful healing of implants with absence of marginal inflammation was encouraging. Moreover, the findings of Periotest system further corroborate our results. Periotest has been used extensively for evaluation and quantification of osseointegration in dental implants^48^. Additionally, the variations in dimension and design of implants, its material composition, or peri-implant bone density doesnot produce extreme disparity in the PTVs^49^. The results of the *ex-vivo* micro-CT evaluation of peri-implant regions regenerated in response to test and control implants demonstrates early bone integration of test implants owing to its osteogenic nanofibrous coating (Supplementary Fig. 3). Further, corroborating evidence could be inferred from the histophotomicrograph of 4^th^ week study model (Supplementary Fig. 4) as described earlier. Furthermore, to minimize the possible biases in the study with low nos. of rabbit models, the predetermined sites for surgical placement of implants for each tibia were meticulously selected after detailed pre-operative radiographic evaluation leading to identification of sites that were roughly equivalent for all rabbit study models.

The occurrence of hyperplastic fibrous mass surrounding the control implants could be due to interfacial micromotion of control implants^50–53^. However, in case of test implants, we presume that due to nanofibrous coating, the increased girth of implants exhibited improved primary stability upon surgical placement resulting in exclusive proliferation of regenerated osseous tissues. Further, a linear relationship between ultimate pullout force and the extent of displacement was noted between test and control implants. The cohesive fracture of bone, far from the surface of test implant strengthen our hypothesis. In *in vivo* physiological environment, the cellular response is determined by the protein adsorbed on the surface of biomaterial^25,54^. Thus, osseointegration of implants depends upon the interplay of biomolecules mainly proteins and the surface of implants^55^. We recognize the fact that inspite of relative standards of numerous biomechanical tests of osseointegrated implants remains contentious, a variety of claims are made regarding the correlation between bone integration with surface roughness and wettability etc. In our previous work we have compared and documented the RMS value and mild hydrophilic features of our coated implant with control and its favourable bio-response^24^. Additionally, the histological and histomorphometric analysis in our present study demonstrated extensive, mature bone tissue formation with higher BIC % around the test implant, post implantation. No bone graft rejection or inflammatory reaction at the interface was noted. The results revealed that the coating is bioactive and osseointegrative. Notably, the gradual disappearance i.e. dissolution of osteogenic nanofibrous coating adds to its advantage of negating the incidence of permanent chronic foreign body reaction and compliments similar studies^56^.

Surfaces with texture like random patterns with pores similar to ours are often associated with increased cell activity and secretion of autocrine/ paracrine regulatory factors^57,58^. Presently, there are no clear understanding on exact and detailed *in vivo* mechanism of osteogenic nanofibres induced bone regeneration. However, previous related studies demonstrate the capability of MSCs in secreting potent combination of trophic factors that modulates either itself (autocrine) or the molecular constituents of the local micro environment in generating favourable bio-response from the neighbouring cells (paracrine effect)^44,59^. Hamidouche *et al*. reported that dexamethasone induced osteoblastic differentiation by upregulating autocrine FGF 18 expression in MSCs^60^. Researchers also claimed that osteogenesis in MSCs was induced by dexamethasone incorporated in synthetic polymer like PLGA acts via autocrine/paracrine pathway^61^. Moreover, ascorbic acid and beta-glycerophosphate are known to involve autocrine/paracrine loop for osteoblastic differentiation^62^. Recently, it was reported that, hydroxyapatite might act in autocrine/paracrine fashion to exert osteoinductive effects on MSCs via the secretion of IL-1a for enhancement of MSCs based bone regeneration^63^. Thus, the precise evaluation of the all the results taken together suggests that at least partially, the enhanced osseointegration may be due to the consequences of cascading effect related to surface influenced endogenous events. Thus, in other words, we can state that the specific bio-response of our osteogenic nanofibrous coated titanium implant surface may be due to consequence of cytokines and molecular messengers secreted by complex autocrine/paracrine regulatory network of MSCs (Fig. 7C) as described earlier in similar or related studies^64–66^.

Of late, it became increasingly apparent that intraosseous implants emerged as an acceptable treatment modality for surgical management of cranio-maxillofacial and orthopaedic cases of various etiology. To identify the ideal implant surface with optimal dynamics, implants have been modified by a plethora of techniques^31,67,68^. However, each modified surface has its unique sets of limitation^69–73^. In 1976, A.E. Clark and colleagues stated that “an ideal implant material must have a dynamic surface chemistry that induces histological changes at the implant interface which would normally occur if the implant were not present”^74^. Liviu Feller *et al*. in their review paper mentioned that “an ideal implant surface should exhibit both osseoconductive and osseoinductive properties, promoting peri-implant bone wound healing and consequently the formation of well-organized mature bone of high mineral and trabecular density with a high proportion of bone-to-implant contact, which will withstand the stress generated on the osseointegrated implant by occlusal forces”^8,25,26^. With our rigorous research efforts, we were successful in fabricating an osteogenic nanofibrous coating on the surface of titanium implant for promotion of osseointegration and the *in vitro* results of which are in close agreement with earlier published reports^24,75,76^. Hence, the end points predicted by our hypothesis closely agrees with our experimental results. Although the exact *in vivo* mechanism involved for such occurrence is yet to be discovered, but due to narrow scatter plot of data in relation to sample size, we can claim with conviction regarding the existence of strong correlation between osteogenic nanofibrous coated titanium implant and its improved osseointegration.

## Conclusion

Our study provides evidence of stronger and favourable bone bio-response resulting in noticeable promotion of osseointegration with respect to osteogenic nanofibrous coated titanium implants, when compared to metallic implants available commercially.

## Methods

### Fabrication of osteogenic nanofibrous coating on the surface of titanium implants

The test implants (Lynx, Equinox, Netherlands) of size 2 mm diameter X 5 mm length were coated with osteogenic nanofibres composed of composite blend of polycaprolactone 5.0% (w/v) (Sigma Aldrich, USA), gelatin type A 0.5% (w/v) (Sigma Aldrich, USA), dexamethasone 0.032% (w/v) (HiMedia, India), β-glycerophosphate 0.5% (w/v) (HiMedia, India), ascorbic acid 0.04% (w/v) (HiMedia, India) and hydroxyapatite 0.04% (w/v) (Budenheim, Germany) in 2,2,2-Trifluoroethanol (Sigma Aldrich, USA). Uncoated titanium screw type implants were used as controls. The fabrication of osteogenic nanofibrous coating on the surface of the implants by electrospinning technique was performed by placing the titanium screw (attached to the rotating shaft of DC motor) in between the syringe tip and collector plate (Fig. 2). The modification of electrospinning apparatus for fabricating nanofibrous coating and its subsequent physico-chemical characterization has been described in detail in our previous study^24^.

### Surface characterization of implants: ESEM and FIB-SEM analysis

The nanofibrous coated and uncoated titanium implant screws were evaluated using an FEI Quanta 200, operating under high-vacuum conditions at an accelerating voltage of 0.7–30 kV. Prior to imaging, all samples were sputter coated with platinum using Auto fine coater JFC-1600 (JEOL, Japan), utilizing 10 mAmps current for 250 sec. Further, FIB-based approach was used for cross-sectional imaging of nanofibrous coated and uncoated implants. Briefly, in the respective area of interest, a protective platinum coated region was milled with Focused-ion beam (FIB) device system (Auriga compact, Carl Zeiss, München, Germany). The FIB device utilizes a 30 kV Ga^+^ ion beam and equipped both with anion, an electron gun (dual beam) and an omniprobe nanomanipulator, allowing *insitu* lift-out of 100 nm thick TEM specimen.

### Animals and surgical procedure

The research protocol was reviewed and approved by the panel of ethics committee for laboratory animal research of ACTREC, Navi Mumbai WIHC/3596—TMH-IITB-ACTREC (Animal study proposal no. 03/2016) and was performed as per the guidelines of the Committee for the Purpose of Control and Supervision of Experiments on Animals (CPCSEA), Ministry of Social Justice and Empowerment, Government of India. The methodology for the present study is as per International Standard ISO-10993-6^77^.

Six healthy adult male New Zealand White rabbits were used in the study (Supplementary Tables 1–3). The popliteal lymph nodes of one additional rabbit (i.e. rabbit without any surgical intervention) was used purely for comparative study to those of study models (n = 6) with conventional H&E staining for standard histological examinations^78^ (Supplementary Fig. 5). All animals were obtained from Reliance Life Sciences Pvt. Limited after due approval from IAEC. Each rabbit was numbered with indelible ink in left pinna and were kept in cages under standardized conditions^79^. The animals were kept on a 12-hour day/night cycle with *ad libitum* access to food and water. Prior to surgery, the osteogenic nanofibrous coated and uncoated implants were sterilized by irradiating with 25 KGy of gamma exposure for 36 hours in the Tissue Bank, Tata Memorial Hospital, Mumbai, India.

Implant surgeries were performed under general anaesthesia induced by ketamine (35 mg kg^−1^) and xylazine (5 mg kg^−1^). The surgical sites for each rabbit were prepared by clipping the fur on bilateral hind limbs, cleaned and disinfected with povidone–iodine. Further, to nullify inter-animal variability like age related variations and weight we have chosen contralateral tibia for implanting the controls. During the surgery, the animals were immobilized, and a longitudinal incision was made on the medial side of the limb and the subcutaneous tissue was dissected all the way down to the bone.

Implant sites were prepared by sequential drilling. First, 1 mm round bur was used to gain access through the cortical bone followed by 1 mm and 2 mm straight drill. All drillings were done under copious saline irrigation to avoid heat generation and resultant adjoining bone necrosis. Implants were placed by hand ratchet with controlled torque to avoid pressure necrosis and achieve initial primary stability (Supplementary Video). Two implants were placed in each tibia with minimal distance of 1 cm. The space provided good blood supply to bone surrounding implant, prevented fracture and resultant implant loss, sufficient margin for resection and histo-morphological slide preparation. The surgical site was closed with multilayer suturing. Subcutaneous tissues were closed with 3.0 absorbable Vicryl suture while skin with 3.0 non absorbable silk suture.

Each rabbit (n = 6) received 4 implants in its tibia - 2 osteogenic nanofibres coated implant in left tibia and 2 uncoated titanium implant in right tibia, respectively. Post-operative radiograph of rabbit’s tibia were obtained immediately to check the position of implants. Later, rabbits were transferred to their respective pens and closely observed for food, water habits, activities and weight gain. Post-operatively, antiseptic Povidone-Iodine ointment was applied on surgical wounds until satisfactory healing. Antibiotics and analgesics Enrofloxacin (5 mg kg^−1^) and buprenorphine (0.03 mg kg^−1^) was administered for 5 days. Further, the rabbits were put in individual cages during the recovery period. Additionally, pre-operative and post-operative radiograph, blood biochemistry and hemogram were evaluated to rule out complication(s) and infections (see Supplementary Information). A score of “0” i.e absence of pain or discomfort^80^ was assigned for all rabbits in accordance with the “Rabbit Grimace Scale” before and post-operative recovery period^80^. Post-surgery, after the completion of respective study periods, the rabbits were euthanized with intravenous administration of Thiopental 100 mg ml^−1^.

### Periotest for assessment of osseointegration

The periotest device (Periotest M, Medizintechnik Gulden, Germany) equipped with a hand piece and tapping rod was used to deliver rapid load (16 times in 4 sec, at a velocity of 0.2 ms^−1^) on the surface of osseointegrated implants. The device calculates the deflection time between the implant and tapping rod into a scaled number ranging from -8 (low mobility) to 50 (high mobility). Lower negative values represent significant osseointegrated implant(s)^81^.

### Macroscopic evaluation and grading

For better objective assessment related to the physiology of implant osseointegration a comprehensive grading system is essential. Further, we believe that the quantitative scoring of implant bone integration will yield vital statistics to establish the correlation between implant osseointegration and its novel coating described herein. The success criteria of implant osseointegration has evolved over the years^82^. Based on the literature, several classification of implant osseointegration, especially of dental implants was found^83–86^. Hence, after deriving the concepts of numerous classifications, a new grading system customized specially for grading implant osseointegration in proof of concept trial in animal models, based on the images and radiograph of rabbit’s tibia, obtained from the present study has been formulated (Table 2). The basic structure of the new macroscopic grading system consists of five necessary variables: “appearance”, “discolouration”, “hyperplastic growth” “peri-implant radiolucency” and “movement of implant”. The variables are meticulously selected and bestowed with equal individual weightage as exacerbation of any of these features alone affects the treatment outcome and success criteria of implants^87^.

**Table 2:** Macroscopic grading system adapted for implant osseointegration in proof of concept trial in animal models.

Parameter	Variables	Score
Appearance of peri-implant region	Normal	0
	Slight surface irregularity	1
	Moderate surface irregularity	2
	Severe disruption/cracks or irregularities	3
	Irregularity with associated complications like suppuration, pus, abscess etc.	4
Discolouration of the implant bone interface with no movement of implant.	Absence of discolouration	0
	Slight discolouration	1
	Moderate discolouration	2
	Severe discolouration	3
	Discolouration with associated complications like suppuration, pus, abscess etc.	4
Presence of distinguished surface mass /hyperplastic growth at the implant bone interface	Absence of hyperplastic growth	0
	Slight hyperplastic growth	1
	Moderate hyperplastic growth	2
	Severe hyperplastic growth	3
	Hyperplastic growth with associated complications like suppuration, pus, abscess etc.	4
Peri-implant bone radiolucency	Absence	0
	Slight	1
	Moderate	2
	Severe	3
Movement of implant	Absence	0
	Slight	1
	Moderate	2
	Severe	3

The physical surface examination of the skin (Fig. 4A), subcutaneous tissue (Fig. 4B) and after dissection of muscles and deep fascia (Fig. 4C) including palpation of popliteal lymph nodes were carried out during necropsy procedure. The implant and peri-implant zone was carefully observed for any implant induced gross morphological or pathological changes. The images of tibial–implant bone complex were recorded by using a Nikon B500 Camera.

The grading scale consists of 5 major parameters with multiple subsets of variables representing well defined clinical conditions. A total score of 0 and 18 represents best and worst possible outcomes respectively.

### Micro-CT evaluation

The *ex-vivo* micro-CT images of each tibia of rabbit (n = 12) containing test and control implants were obtained by using a micro CT imaging system (Gamma Medica-Ideas, FLEX™ Triumph™ Pre-Clinical Imaging System, Northridge, CA, USA) by scanning along the long axis of the specimen to compare the bone mineral density (BMD) and bone mineral content (BMC) of peri-implant area. The micro-CT measurement was performed with a voltage of 40 KeV with exposure time of 600 ms. The focal spot was 84 μm with 2X magnification having field of view (FOV) 59.2 mm. For calculation of Implant Bone Integrated volume (IBIV)^24^, an anatomical contour resembling roughly a rectangle over the diaphyseal trabecular bone adjacent to the surface of implant was considered as volume of interest (VOI) over two subsequent stacks (Supplementary Fig. 2). The manual region of interest (ROI) included zone of mineralized tissue ~1.3 mm distal from the long axis of the implant. A constant VOI was maintained with respect to size and shape for both test and control implants. The data was observed and analysed by GE MicroView CT program and OsiriX software respectively.

### Pull-out test

The osseointegrated implants (n = 3 + 3) were subjected to biomechanical evaluation. The tibiae of rabbit with corresponding osseointegrated implants were subjected to uniaxial tensile measurement using an Instron testing machine (QC 3345, Norwood, MA, USA). The ultimate tensile strength as defined for our experiment is the peak force required to completely displace the implant upwards from the bone. The upper part of the screw head was attached to the fixture arm of the testing machine by a mechanical chuck while the proximal end of the bone was fixed on the platform. The pull out test (n = 3 + 3) was accomplished by setting a distraction rate of 0.5 mm min^−1^ with a longitudinal force directed to the long axis of the implant and the measurements were recorded on a force versus displacement plot. The force vs displacement curve were continuously registered until a clear drop in the pull-out force was noted.

### Histological evaluation

After euthanizing the rabbits with high dose of thiopental injection, the tibiae along with implants were removed and fixed in 10% neutral buffered formalin (Sigma Aldrich, USA). Subsequently, the formalin fixed tissue – implant blocks were dehydrated in graded series of ethanol and embedded in plastic resin (Methyl- Methacrylate, Loba Chemie, India) for non-decalcified sectioning. A diamond coated saw (Buehler, IsoMet LS Precision Sectioning Cutter, USA) was used to section the resin embedded implant in bone into two equal parts. The samples were then gradually grinded and polished using silicon carbide grinding papers (Struers, Germany) until a thickness of 90–110 μm were obtained. All the sections were stained with haematoxylin (HiMedia, India) - eosin (HiMedia, India), and examined under optical microscopes (Stereozoom microscope LM-52-3611, Lynxinst, India and Olympus BX53M, Olympus Corp., Tokyo, Japan) for analysis of regenerated osseous tissue. Bone-to-implant contact was measured by dividing the bone contact perimeter by the total perimeter of the implant^88^.

### Statistics

All numerical data were expressed as mean ± standard error of mean (SEM). Statistical analysis was performed using OriginPro software (OriginLab Corporation). Obtained data sets were first subjected to Kolmogorov-Smirnov test to check their distribution. Accordingly, Student’s t-test was performed for parametric data sets and *P* < 0.05 was considered statistically significant.

## Supplementary information

Surgical placement of ONFC implant

Supplementary Information

## Data Availability

All data generated or analyzed during this study are included in this published article and its supplementary. Raw data and additional details can be provided on request.
