# Supplementary material for: Accentuated osseointegration in osteogenic nanofibrous coated titanium implants
Source: Sci Rep. 2019 Dec 9;9:17638. doi: 10.1038/s41598-019-53884-x (PMC6901521; doi:10.1038/s41598-019-53884-x)
Supplement: Supplementary file 2 — Supplementary Information [file 41598_2019_53884_MOESM2_ESM.pdf]

Accentuated osseointegration in osteogenic nanofibrous coated titanium implants

Siddhartha Das[1,2†], Kanchan Dholam[3†], Sandeep Gurav[3†], Kiran Bendale [4†], Arvind Ingle [4†], Bhabani Mohanty [4†], Pradip Chaudhari[4] and Jayesh R. Bellare[2,5*]

[1]Department of Biosciences and Bioengineering, Indian Institute of Technology Bombay, Mumbai - 400076, Maharashtra, India

[2]Department of Chemical Engineering, Indian Institute of Technology Bombay, Mumbai - 400076, Maharashtra, India

[3]Department of Dental and Prosthetic Surgery, Tata Memorial Centre, HBNI, Mumbai- 400 012, Maharashtra, India

[4]Advanced Centre for Treatment, Research and Education in Cancer, Navi Mumbai, - 410 210, Maharashtra, India

[5]Wadhwani Research Centre for Bioengineering, Indian Institute of Technology Bombay, Mumbai, - 400076, Maharashtra, India

[†]The first six authors contributed equally to this work.

*Corresponding Author : Prof. Jayesh R. Bellare

E-mail: jb@iitb.ac.in

Contact number: +91 (22) 2576 7207 (O), +91 (22) 2572 6895 (Fax)

Supplementary Information

Table of Contents

Supplementary Figures

Supplementary Figure 1	*PTVs of test (T) and control (C) implants in rabbits……………………………………2*
Supplementary Figure 2	*Length of cortical bone adjacent to implants & Implant Bone Integrated Volume (IBIV)…………………………………………………………………………………3*
Supplementary Figure 3	*Representative micro-CT images of implants in tibia of rabbit study models………………………………………………………………………………………4*
Supplementary Figure 4	*H and E stained interfacial tissue of test implant 4 weeks after implantation………………………………………………………………………………….5*
Supplementary Figure 5	*Histology of popliteal lymph nodes of 12th week rabbit study models………………………………………………………………………………………9*

Supplementary Tables

Supplementary Table 1	*Weight (Kg) of rabbits during the study period…………………………………………6*
Supplementary Table 2	*Blood Biochemistry………………………………………………………………………………7*
Supplementary Table 3	*Hemogram…………………………………………………………………………………………8*

Supplementary movie caption ……………………………………………………………………………………10

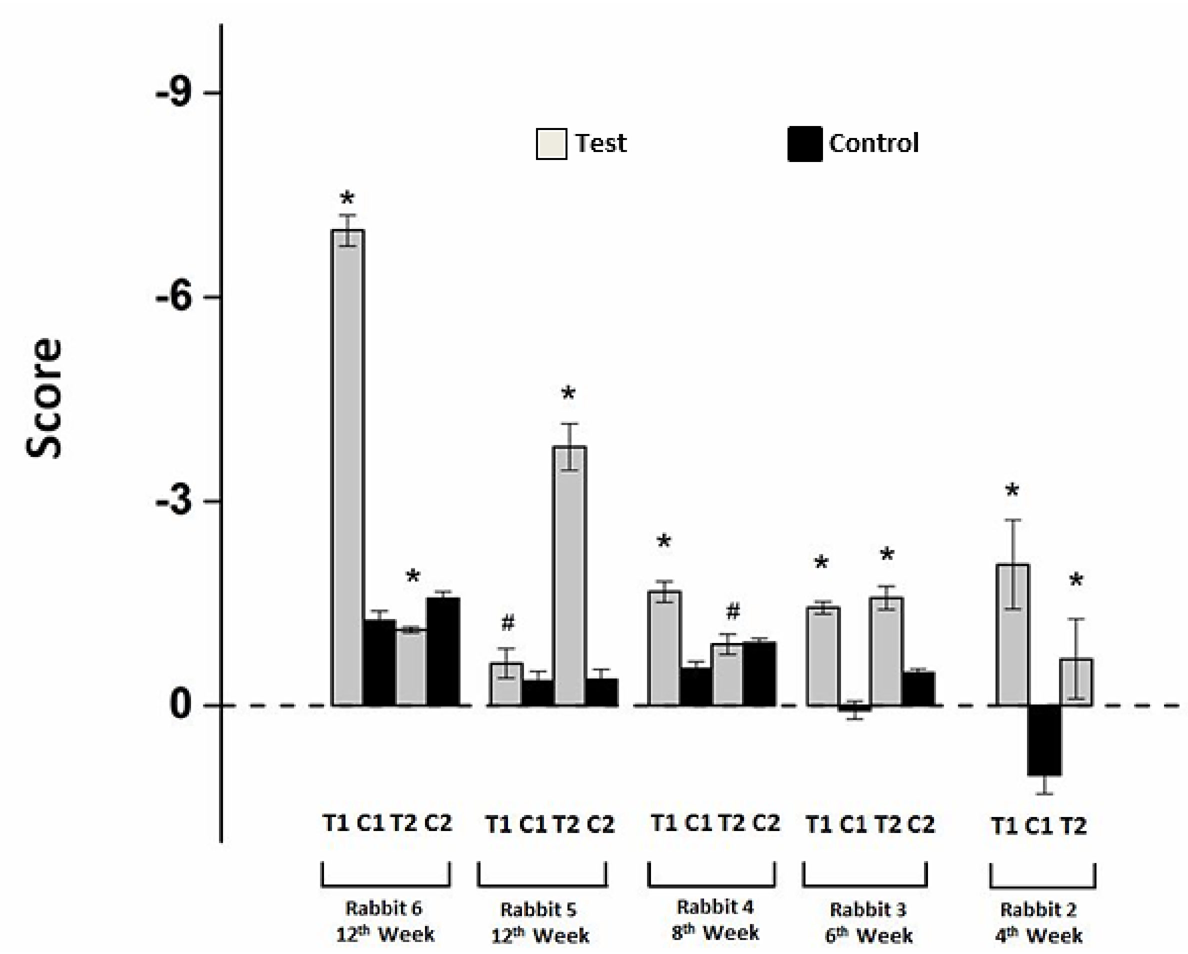

Supplementary Figure 1: *Periotest values (PTVs) of test (T) and control (C) implants in rabbits.* PTVs of test implants were found to be more negative than controls. The magnitude of values for test implants decreases gradually suggesting a progressive increase in implant stability/osseointegration over the study period when compared to controls. Abbreviations: - T1 - Test implant no. 1, - T2 -Test implant no. 2, - C1 - Control implant no. 1, - C2 - Control implant no. 2. Data are expressed as mean ± standard error of mean (SEM). Student's t-test was performed with data significance indicated with (*) for $p < 0.05$.

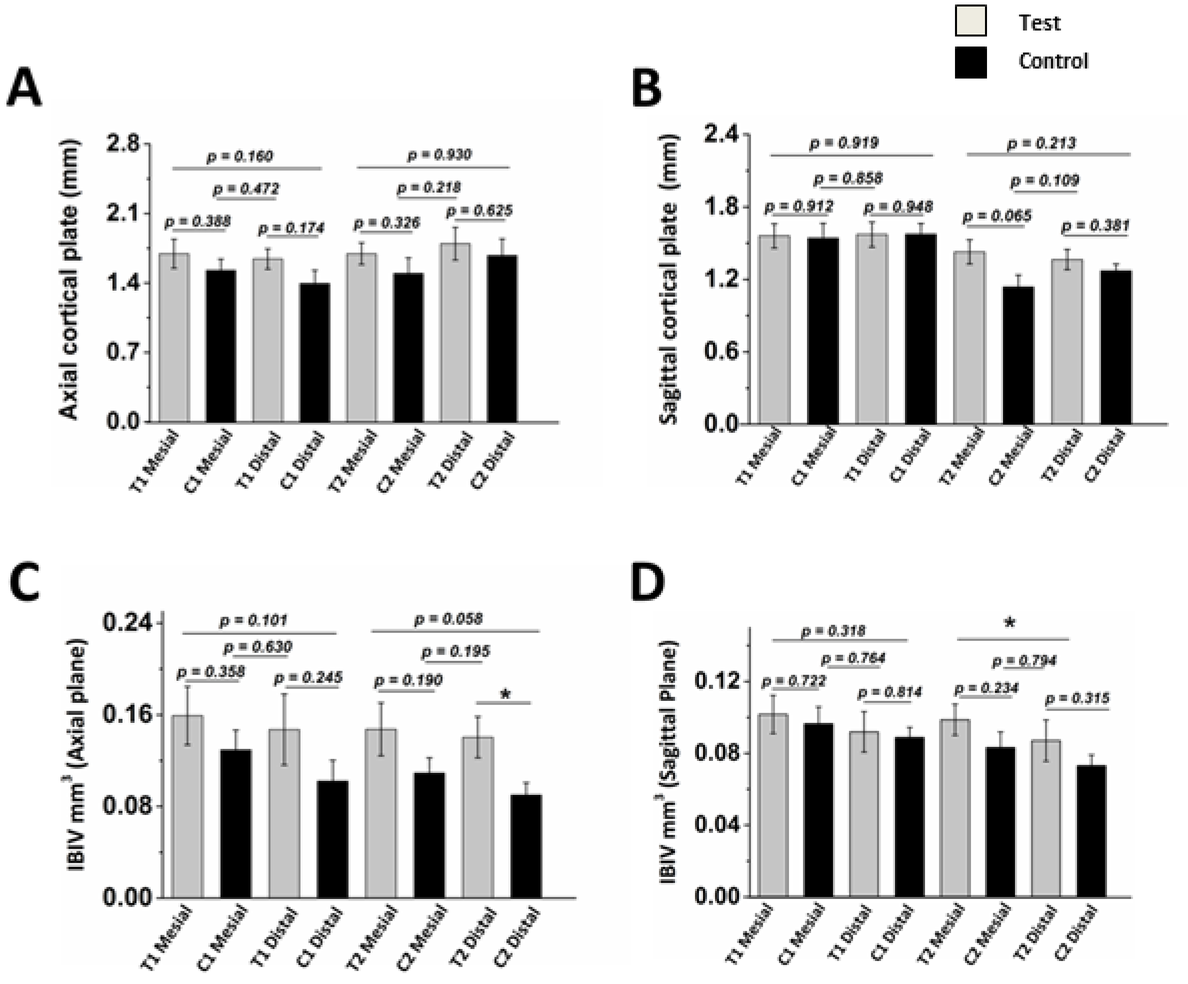

Supplementary Figure 2: *Length of cortical bone adjacent to implants in (A) axial plane, (B) sagittal plane (C) Implant Bone Integrated Volume (IBIV) in axial plane, (D) Implant bone integrated volume (IBIV) in sagittal plane***.** Test implants demonstrated improved results compared to controls. Abbreviations: - T1 - Test implant no. 1, - T2 -Test implant no. 2, - C1 - Control implant no. 1, - C2 - Control implant no. 2. Data are expressed as mean ± standard error of mean (SEM). Student's t-test was performed with data significance indicated with (*) for $p < 0.05$.

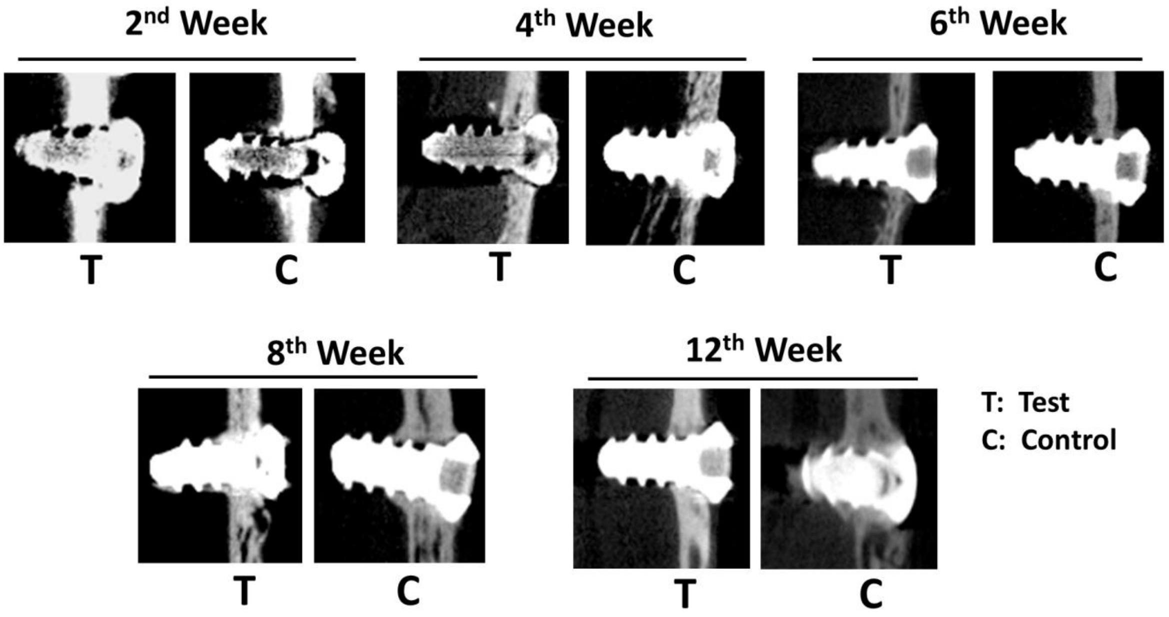

Supplementary Figure 3: *Representative micro-CT images of implants in tibia of rabbit study models.* 2D mico-CT images of test implants demonstrate early bone integration compared to respective controls.

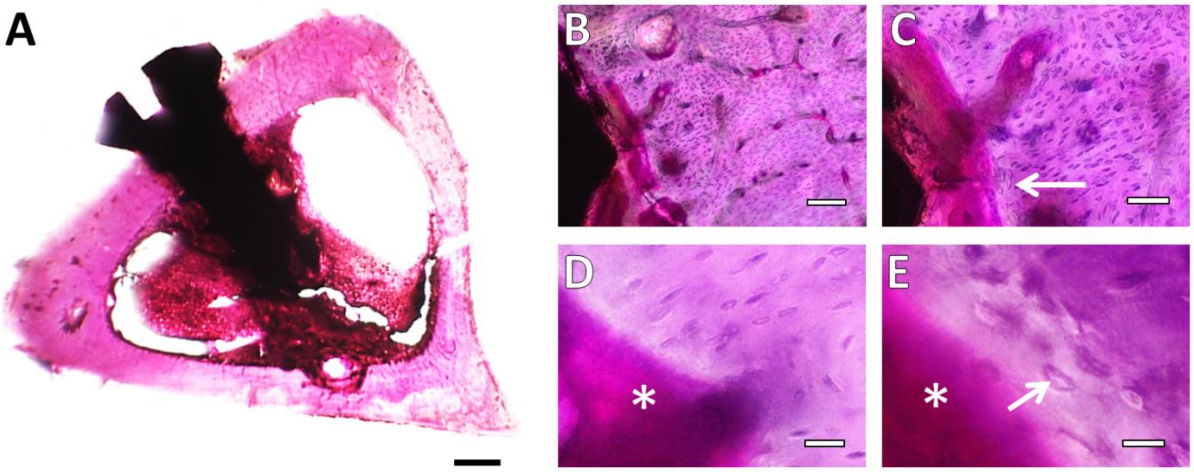

Supplementary Figure 4: *H and E stained interfacial tissue of test implant 4 weeks after implantation.* Mature bony architecture is observed far from the interfacial region. Active osteoblasts (↑) juxtaposed to osteoid seam (*) is noted. Scale bar for A represents 1 mm, scale bar for B represents 100 µm, scale bar for C represents 50 µm, scale bar for D represents 20 µm and scale bar for E represents 10 µm.

Supplementary Table 1: Weight (Kg) of rabbits during the study period.

Date	Rabbit 1	Rabbit 2	Rabbit 3	Rabbit 4	Rabbit 5	Rabbit 6	Rabbit 7
10-11-2017	3.00	2.74	2.85	2.18	2.87	2.50	2.72
17-11-2017	3.00	2.52	2.99	2.33	2.95	2.61	2.94
07-12-2017	3.06	2.95	3.06	2.49	3.02	2.61	
27-12-2017	3.29	3.25			3.29	2.77	3.36
01-01-2018	2.76				3.38	2.83	3.43

No significant difference in reduction or increases in body weight of rabbits were noted.

Supplementary Table 2: Blood Biochemistry

Animal	Date	BUN	CREAT	TP	ALB	ALP	TBI	AST	ALT
Rabbit 1	22-11-2017	52	1.78	6.4	1.76	161	0.6	83	118
	17-01-2018	55	0.86	5.6	1.69	70	0.2	27	56
Rabbit 2	22-11-2017	54	1.24	6.7	1.71	180	0.9	58	20
	20-12-2017	64	1.23	5.5	1.67	150	0.2	41	51
Rabbit 3	22-11-2017	8	1.51	6.2	1.64	136	0.4	104	48
	03-01-2018	56	0.87	5.4	1.67	124	0.3	44	42
Rabbit 4	29-11-2017	55	1.08	5.8	1.75	189	0.2	31	72
	13-12-2017	2	0.47	5.4		126	0.2	21	48
Rabbit 5	29-11-2017	53	1.19	5.7	1.65	109	0.3	34	31
	19-02-2018	51.9	1.12	6.11	1.81	103	0.3	36	27
Rabbit 6	29-11-2017	50	1.34	6.1	1.91	114	0.3	39	67
	19-02-2018	61.2	1.21	5.55	1.73	101	0.3	42	60
Rabbit 7	19-02-2018	49.3	1.12	5.33	1.55	45	0.3	73	57

BUN: Blood urea nitrogen

CREAT: Serum creatinine

TP: Total protein

ALB: Serum albumin

ALP: Alkaline phosphatases

TBI: Total bilirubin

AST: Aspartate aminotransferase

ALT: Alanine aminotransferase

Supplementary Table 3: Hemogram

Animal	Date	Hb	RBC	WBC	PCV	PLT
Rabbit 1	22-11-2017	15	6.55	1.42	50.8	160
	17-01-2018	14.6	6.33	4.83	48.4	159
Rabbit 2	22-11-2017	12.6	5.15	2.48	47.8	180
	20-12-2017					
Rabbit 3	22-11-2017	14	6.26	1.80	50.1	301
	03-01-2018	12.6	5.69	3.45	40.8	351
Rabbit 4	29-11-2017	14.5	6.61	5.35	46.2	243
	13-12-2017	13.5	6.10	4.77	44.5	178
Rabbit 5	29-11-2017	12.8	5.90	6.73	42.9	207
	19-02-2018	14.2	6.61	1.06	46.5	295
Rabbit 6	29-11-2017	13.6	5.78	5.64	44.5	270
	19-02-2018	13.9	6.32	1.74	46.2	291

Hb: Hemoglobin Concentration

RBC: Red Blood Cell

WBC: White Blood Cell

PCV: Packed Cell Volume

PLT: Platelet Blood Test

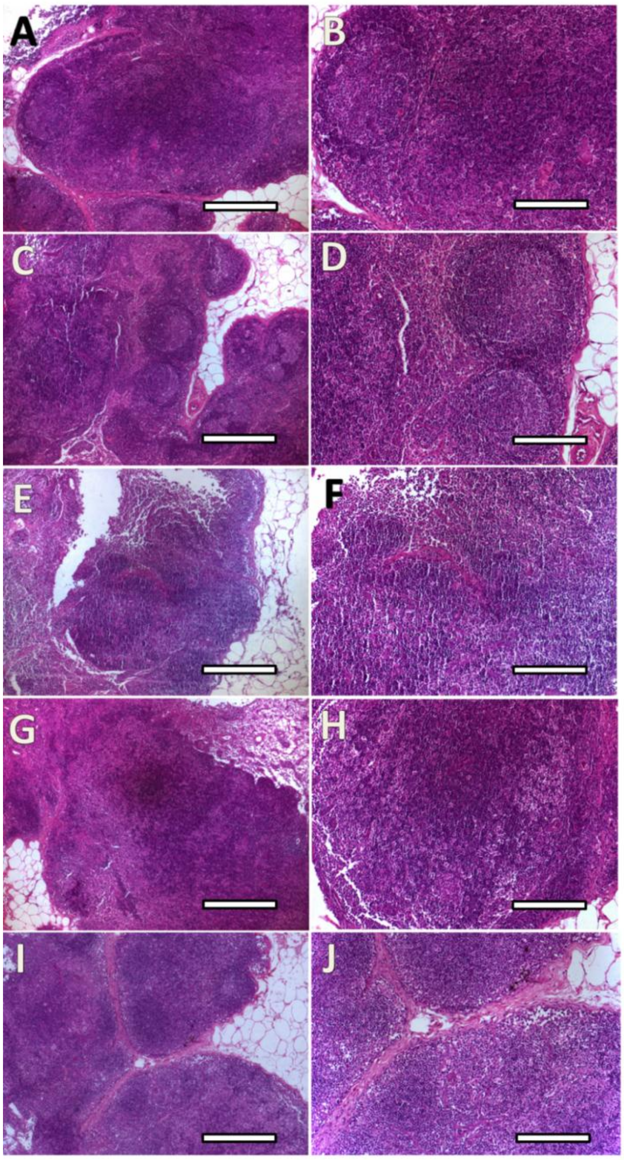

Supplementary Figure 5: *Histology of popliteal lymph nodes of 12th week rabbit study models.* Normal features of lymph nodes like lymphoid follicles, germinal centres, cortex and medulla etc. are noted. No histological abnormalities were detected in the stained sections of lymph nodes obtained from controls (A, B, E and F), tests (C, D, G and H) and additional rabbit (I and J) study models. Scale bar for A, C, E, G and I represents 400 µm. Scale bar for B, D, F, H and J represents 200 µm.

Supplementary Movie: *Representative video of surgical placement of coated titanium implants.* The video of the surgical placement of implant shows that the implant along with its intact coating could be placed in bone.
